# Association Between the Treatment Modality of Pediatric Subcondylar Fractures and Functional Outcomes at the Six-Month Follow-Up: A Retrospective Pilot Study

**DOI:** 10.7759/cureus.76226

**Published:** 2024-12-22

**Authors:** Dominik Scheibl, Benjamin Walch, Michael Verius, Carolin Götz, Rüdiger Emshoff

**Affiliations:** 1 Department of Oral and Maxillofacial Surgery, Medical University of Innsbruck, Innsbruck, AUT; 2 Department of Radiology, Medical University of Innsbruck, Innsbruck, AUT

**Keywords:** adolescents, children, classification, closed reduction, cone beam computed tomography, functional outcome, mandible, open reduction, regression analysis, subcondylar fractures

## Abstract

Background: The choice of treatment for subcondylar fractures in children and adolescents remains a controversial issue. The aim of this study was to evaluate the association between the treatment modality of subcondylar fractures and functional outcomes at the six-month follow-up.

Methods: This retrospective study examined a cohort of children and adolescents with unilateral or bilateral subcondylar fractures treated at a level 1 trauma center over a five-year period. Radiological assessments of ramal height shortening (RHS) and subcondylar fracture angulation (SFA) were conducted using cone beam computed tomography. A total of 28 patients met the inclusion criteria, which required participants to be 18 years of age or younger, have a follow-up period of at least six months, and present with an initial SFA of less than 45°. Subcondylar fractures were classified into three categories: Class I (RHS <2 mm and/or SFA <10°), Class II (RHS ≥2 mm and ≤15 mm and/or SFA ≥10° and ≤35°), and Class III (RHS >15 mm and/or SFA >35°). Functional outcomes, including maximal mouth opening, lateral movements, and protrusive movements, were assessed at the six-month follow-up. Reference values specific to children and adolescents were applied to distinguish between normal and limited mandibular motion. The relationship between treatment modality and functional outcomes was analyzed using logistic regression, with adjustments made for age, sex, and fracture classification.

Results: Twenty-eight patients (67.9% male; mean age 14.0±4.0 years) met the inclusion criteria. Of these, 53.6% (n=15) were treated with open reduction and internal fixation, while 46.4% (n=13) underwent closed reduction. The choice of treatment modality significantly influenced patient prognosis. Closed reduction was strongly associated with improved functional outcomes, specifically in the vertical range of movement (odds ratio (OR)=16.4; P=0.047), lateral range of movement (OR=18.7; P=0.044), and overall combined functional outcomes of vertical, lateral, and protrusive movements (OR=10.9; P=0.028).

Conclusion: This preliminary study suggests a correlation between treatment modality and functional outcomes at the six-month follow-up. Open reduction and internal fixation of subcondylar fractures in children and adolescents may carry a higher risk of poor functional outcomes. The findings support closed reduction as the preferred approach for Class I-III cases with subcondylar fracture fragments angulated between 0° and 45°.

## Introduction

Children and adolescents have unique anatomical structures and physiological functions, making the treatment of mandibular fractures distinct from that in adults [1,2]. Condylar process fractures (MCFs) are the most common type of mandibular fracture in this age group, accounting for approximately 56% of all mandibular fractures [3,4]. These fractures are a critical aspect of pediatric facial trauma management, as improper treatment can lead to long-term complications such as facial growth disturbances, mandibular asymmetry or deficiency, malocclusion, ankylosis, restricted mouth opening, and temporomandibular joint (TMJ) dysfunction [3-5].

While studies have shown that both surgical and conservative approaches can yield satisfactory outcomes for displaced MCFs in children and adolescents [3,6,7], the optimal management strategy remains a subject of debate. Surgical treatment is often suggested for severely displaced or dislocated fractures, while conservative management may be suitable for less severe cases involving minimal displacement or a minor reduction in ramal height. Factors such as the extent of injury (unilateral or bilateral), fracture level, dental malocclusion, mandibular dysfunction, age, dentition status, concomitant facial injuries, the patient's overall condition, and the surgeon's experience also play a role in determining the treatment approach [7,8].

Relying on panoramic radiography for clinical decision-making may be problematic due to the potential for distortion and superimposition of adjacent structures, leading to inaccurate diagnoses and inappropriate treatment [9,10]. In contrast, modern computed tomography (CT) imaging modalities, including helical multidetector CT and cone beam CT (CBCT), offer detailed, accurate visualization of the fractured subcondyle [11,12]. CBCT provides precise orientation and angulation data with significantly reduced radiation exposure, up to 25 times lower than multidetector CT, while being less affected by beam-hardening artifacts [13,14]. Multidetector CT, however, excels in soft-tissue contrast, allowing better characterization of fracture fragments, their relationship to surrounding structures, and associated soft-tissue injuries [13]. 

Despite advancements in imaging, a consensus on classification schemes and treatment protocols for MCFs in children and adolescents remains elusive. Clinicians often rely on personal experience or institutional guidelines [15,16]. A review of recent literature indicates a general preference for surgical intervention in severely displaced or dislocated fractures and conservative management for less severe cases [3,4,7,17-19]. However, the precise thresholds of displacement and angulation that would favor open reduction and internal fixation (ORIF) over conservative methods remain unclear.

To date, no comparative studies using CT or CBCT have investigated the role of clinical and radiological parameters in defining specific functional outcome criteria for pediatric MCF management in a multivariate design. This pilot study aims to evaluate the relationship between treatment modality for subcondylar fractures and functional outcomes at six months post-treatment while adjusting for variables such as age, sex, and subcondylar fracture classification.

## Materials and methods

Study design, population, and inclusion and exclusion criteria

The medical records of pediatric patients diagnosed with mandibular fractures and treated at the Clinic of Oral and Maxillofacial Surgery at the University of Innsbruck from January 2014 to April 2019 were retrospectively reviewed. Informed consent was obtained from all adult participants as well as the parents or guardians of the children included in the study. The research adhered to the principles outlined in the Declaration of Helsinki regarding medical ethics and protocols and received approval from the Institutional Review Board (IRB) at the Medical University of Innsbruck (approval number: IMU/IRB/1153/2018).

**Figure 1:**
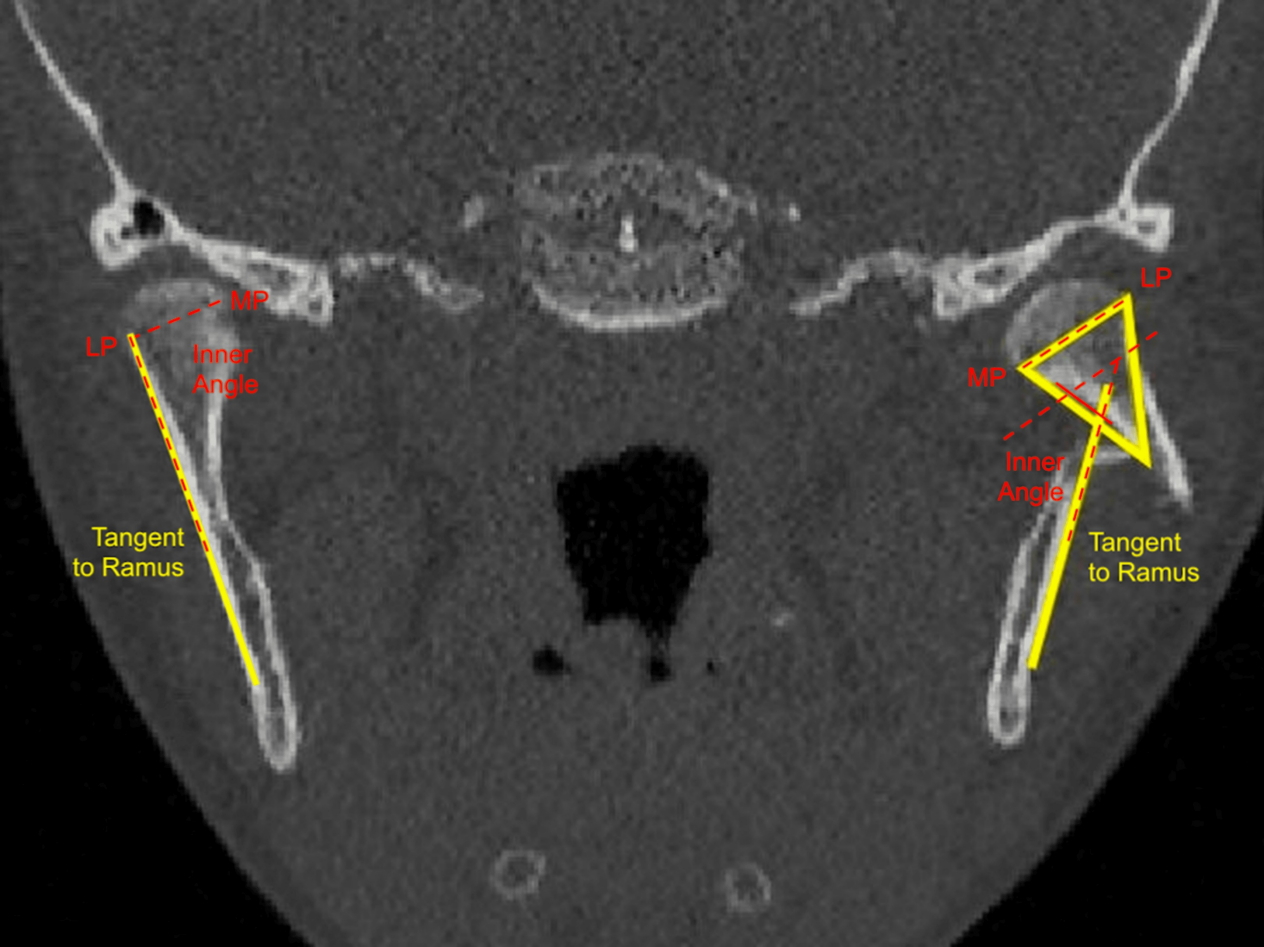
Condylar displacement is quantified on coronal CBCT image using the Vidhya et al. method A line is drawn between the medial and lateral poles of the condyle. Another line is drawn tangent to the ramus. The inner angle formed by the intersection of the two lines is calculated. The difference between the angles on the non-fractured and the fractured sides is used as a measure of coronal displacement. CBCT: cone beam computed tomography; LP: lateral pole; MP: medial pole

Patients were included in the study if they were 18 years old or younger, had unilateral or bilateral mandibular condylar fractures confirmed by CBCT, had bilateral fractures managed conservatively, and exhibited a subcondylar fracture angulation (SFA) of 45 degrees or less as assessed on CBCT coronal scans (Figure 1).

Additionally, their fractures had to be managed, either conservatively or surgically, within five days of injury, and they needed to have complete clinical records available at the six-month follow-up.

Exclusion criteria included the presence of systemic inflammatory diseases such as idiopathic arthritis, a history of prior orthognathic surgery, pre-existing temporomandibular joint (TMJ) disorders, concomitant facial fractures, or fractures previously treated surgically at other institutions.

CBCT data acquisition and subcondylar fracture classification

A CBCT machine (KaVo 3D eXam; KaVo Dental GmbH, Biberach, Germany) was utilized to evaluate condylar morphology. The entire condylar head, including axial, coronal, and sagittal views, was assessed using CBCT imaging. During the scan, patients were positioned upright with their teeth in centric occlusion, and the Frankfort horizontal plane aligned parallel to the floor. The scanning parameters were set to a 16×13 cm field of view, 90 kVp tube voltage, 8.0 mA tube current, and a 24-second scan time. The acquired data were reconstructed using OnDemand3D software (KaVo Dental GmbH, Biberach, Germany) for three-dimensional analysis.

CBCT imaging was performed on patients with unilateral or bilateral subcondylar fractures. Based on the CBCT images, unilateral subcondylar fractures were classified into three categories following the method described by Bhagol et al. [20]: Class I (minimally displaced) with ramal height shortening of less than 2 mm and/or fracture displacement of less than 10°; Class II (moderately displaced) with ramal height shortening between 2 and 15 mm and/or fracture displacement between 10° and 35°; and Class III (severely displaced) with ramal height shortening exceeding 15 mm and/or fracture displacement greater than 35°.

For unilateral fractures, the degree of displacement and ramal height shortening was evaluated using the method outlined by Vidhya et al. [21]. In cases of bilateral mandibular condylar fractures, the more severely displaced fracture site was used for categorizing the fracture class, as described by Chang et al. [22].

Treatment and follow-up

Conservative treatment for MCFs varied based on the patient's age and the degree of fracture displacement. This approach included a soft diet combined with active mouth-opening exercises, guided occlusion using a removable orthodontic appliance, and maxillomandibular fixation (MMF) with elastics for a duration of 7-42 days.

Surgical treatment of MCFs was performed using the retromandibular approach, with fracture fixation achieved using 2 mm titanium miniplates. Following surgery, MMF with light elastics was maintained for 3-5 days. All patients received postoperative instructions emphasizing mouth-opening exercises and physiotherapy. Associated mandibular fractures were managed with either open reduction techniques, including plate osteosynthesis and screw fixation, or MMF with elastics, depending on the specifics of the injury.

Patients were scheduled for follow-up visits at one, three, and six months after treatment. Clinical and radiographic findings from both the initial assessment and subsequent follow-ups were documented using standardized recording schemes. The accuracy of fracture reduction and fixation stability was evaluated using panoramic radiographs obtained preoperatively, immediately postoperatively, and at the three- and six-month follow-ups. To minimize radiation exposure, posteroanterior radiographs and CBCT scans were not routinely performed.

Evaluation of clinical outcomes

Clinical follow-ups involved a comprehensive orofacial examination, with a particular focus on assessing the mandibular range of motion (ROM), occlusion pattern, and any subjective symptoms related to the TMJ. Stable intercuspidation between the dental arches was considered an acceptable occlusion. During these visits, patients were asked whether jaw opening and movement caused pain, as well as to describe their chewing function as either normal or reduced. Functional parameters were systematically recorded during the first week after treatment, as well as at the one-, three-, and six-month follow-up visits.

Clinical mandibular ROM recording 

The study was conducted following a pre-established, standardized protocol for assessing mandibular mobility [23]. The primary outcome variables were the unassisted mandibular ROM in vertical, lateral, and protrusive directions. Patients were seated in a dental chair, and measurements were taken using a millimeter ruler.

Maximal mouth opening capacity was defined as the interincisal distance between the upper and lower incisors at the maximum opening position, taking into account any existing vertical incisal overlap. Patients were instructed to open their mouths as wide as possible, even if it caused discomfort. The edge of the millimeter ruler was placed at the incisal edge of the upper central incisor, and the distance to the incisal edge of the lower incisor was measured and recorded.

Maximal laterotrusion capacity was defined as the greatest mandibular side-to-side excursion from the full intercuspal position to the most extreme left or right mandibular position, using the dental midlines as reference points. Patients were asked to slightly open their mouths from the physiological rest position and move the mandible as far as possible to the right or left. The movement was measured using a millimeter ruler, from the labioincisal embrasure of the maxillary central incisors to the labioincisal embrasure of the mandibular incisor.

Maximal protrusion capacity was defined as the greatest forward mandibular excursion from the full intercuspal position to the most extreme forward mandibular position, using the maxillary and mandibular central incisors as reference points. Patients were instructed to move the mandible forward from the physiological rest position, ensuring no tooth contact. The distance from the incisal edge of the maxillary central incisor to the incisal edge of the mandibular central incisor was measured. The horizontal overlap was also measured and added to the distance between the upper labial surfaces and the lower incisal edge.

Study variables

The primary predictor in this study was the treatment modality, specifically comparing closed reduction and open reduction. The maximum mandibular ROM in vertical, lateral, and protrusive directions at the six-month follow-up served as the primary outcome measures. Covariates included sex, age, and subcondylar fracture class.

To assess the functional outcomes, distribution-based reference values for children and adolescents were used to distinguish between "limited" and "normal" mandibular motion. Vertical and lateral ROM values were compared to age- and sex-specific percentile norms for healthy children and adolescents [24,25]. Any patient whose ROM was below the third percentile was considered to have below-normal mandibular movement. Protrusive ROM values were compared to age-specific reference ranges, with values taken from studies on healthy pediatric subjects aged 6-9 years [26] and 10-17 years [27].

Statistical analysis

The differences between baseline variables in the study groups were analyzed using the chi-squared analysis for categorical data and an independent sample t-test for continuous data. A logistic regression analysis was employed to evaluate the association between treatment modality and functional outcomes, specifically normal mandibular ROM in the vertical, lateral, and protrusive directions. Data were adjusted to account for covariates such as age, sex, and subcondylar fracture class. Statistical significance was determined with a p-value of less than 0.05. The IBM SPSS Statistics for Windows, Version 28.0 (Released 2021; IBM Corp., Armonk, New York, United States) was used for statistical analysis.

## Results

**Figure 2:**
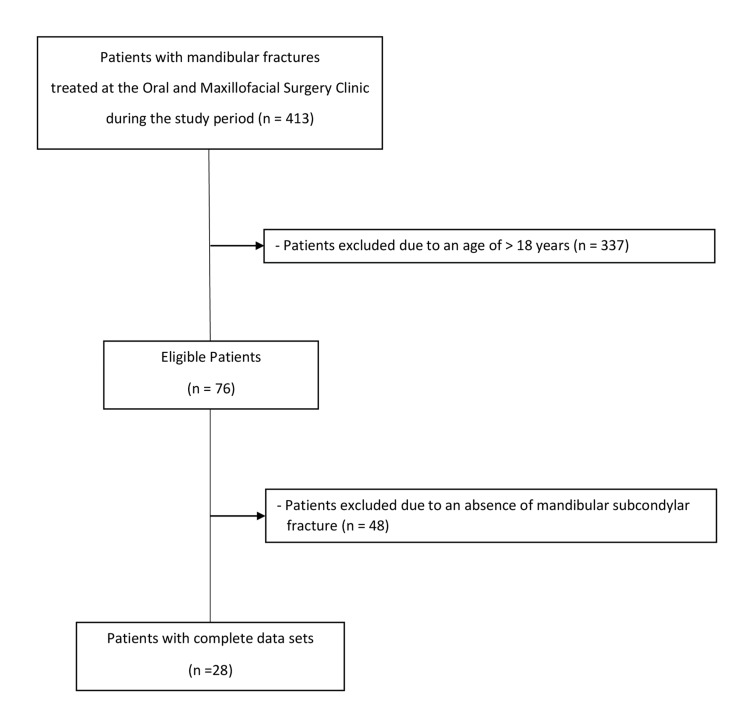
Flowchart demonstrating the selection process of the study population

Out of 413 consecutive patients treated for mandibular fractures, 337 were excluded from the study due to being over 18 years old. Of the remaining 76 patients, 48 were excluded because they did not have an MCF. This left a total of 28 patients with either unilateral (n=23; 82.1%) or bilateral (n=5; 17.9%) MCFs (Figure 2).

Among these patients, 12 (42.9%) had an associated isolated mandibular fracture in the symphysis (n=8; 28.6%) or body (n=4; 14.3%) or a multiple mandibular fracture (n=4; 14.3%). The fractures were caused by various etiologies: sport-related injuries (n=16; 57.1%), assault (n=5; 17.9%), and bicycle accidents and syncope (n=1 each; 3.6%).

The mean age of the patients was 14.0±4.0 years (range: 6-18 years), of whom 67.9% were male. Subcondylar fracture classifications were as follows: Class I in 11 subjects (39.3%), Class II in 11 subjects (39.9%), and Class III in six subjects (21.4%). The open reduction group included 15 patients (10 males, five females; mean age: 15.7±2.3 years), and the closed reduction group included 13 patients (nine males, four females; mean age: 12.2±4.7 years). Among the cohort, 75% (n=21) were over the age of 12, with 67% (n=14) managed by closed reduction. Of the seven patients aged 12 or younger, 57.1% (n=4) were treated with closed reduction. Data analysis revealed a significantly higher mean age in the open reduction group compared to the closed reduction group (15.7 years vs. 12.2 years; P=0.025), though there were no significant differences in subcondylar fracture classes (P=0.701). Furthermore, functional outcomes did not differ significantly between the open and closed reduction groups (P>0.05) (Table 1).

**Table 1:** Patient variables by treatment group n: number of patients; p-value: probability of type I error ^*^Significance at p-value <0.05 using independent samples t-test ^#^Significance at p-value <0.05 using the chi-squared analysis

Variables	Treatment groups			P-value
	Open reduction (n=15)	Closed reduction (n=13)	Total (n=28)	
Baseline variables				
Demographics				
Age (years) (mean±SD)	15.7±2.3	12.2±4.7	14.0 ± 4.0	0.025^*^
Males (n) (%)	10 (66.7)	9 (69.2)	19 (67.9)	
Females (n) (%)	5 (33.3)	4 (30.8)	9 (32.1)	0.058^#^
Subcondylar fracture classification				
Class 1 (n) (%)	5 (33.3%)	6 (46.2%)	11 (39.3%)	
Class 2 (n) (%)	6 (40%)	5 (38.5%)	11 (39.3%)	
Class 3 (n) (%)	4 (26.7%)	2 (15.4%)	6 (21.4%)	0.701^#^
Outcome variables				
Normative value for range of movement				
Vertical (n) (%)	7 (46.7%)	10 (76.9%)	17 (60.7%)	0.102^#^
Lateral (n) (%)	7 (46.7%)	10 (76.9%)	17 (60.7%)	0.102^#^
Protrusive (n) (%)	10 (66.7%)	11 (84.6%)	21 (75%)	0.274^#^
Overall combined (n) (%)	4 (26.7%)	8 (61.5%)	12 (42.9%)	0.063^#^

For each functional outcome variable, regression analyses were performed with treatment modality as the independent variable, adjusting for age, sex, and subcondylar fracture class. The results indicated that closed reduction was significantly associated with improved functional outcomes, specifically in vertical (odds ratio (OR)=16.4; P=0.047) and lateral (OR=18.7; P=0.044) ROM, as well as overall combined normal function in vertical, lateral, and protrusive movements (OR=10.9; P=0.028) (Table 2).

**Table 2:** Binary multivariate regression analysis of predictive factors of normative values for mandibular range of motions at the six-month follow-up n: number of patients; B: regression coefficient; S.E.: standard error; p-value: probability of type I error; OR: odds ratio; CI: confidence interval *Significance at p-value <0.05 using binary multiple regression analysis

Outcome variable	B	S.E.	Wald statistic	P-value*	OR	95% CI
Vertical range of motion						
Age	0.281	0.165	2.901	0.089	1.325	0.958-1.832
Sex	0.138	1.040	0.018	0.894	1.148	0.150-8.810
Fracture class	0.382	0.725	0.277	0.599	1.465	0.353-6.069
Treatment	2.798	1.407	3.953	0.047	16.405	1.041-258.651
Lateral range of motion						
Age	0.283	0.171	2.748	0.097	1.328	0.950-1.856
Sex	-0.476	1.048	0.207	0.649	1.649	0.207-12.549
Fracture class	0.832	0.786	1.120	0.290	2.299	0.492-10.737
Treatment	2.928	1.455	4.050	0.044	18.684	1.079-323.391
Protrusive range of motion						
Age	0.147	0.130	1.290	0.256	1.159	0.899-1.495
Sex	-0.325	0.956	0.116	0.734	0.722	0.111-4.708
Fracture class	0.220	0.623	0.125	0.724	1.246	0.368-4.223
Treatment	0.373	0.980	0.145	0.703	1.452	0.213-9.904
Overall range of motion						
Age	0.216	0.143	2.286	0.131	1.241	0.938-1.642
Sex	-0.510	0.991	0.265	0.606	0.600	0.086-4.184
Fracture class	0.160	0.635	0.064	0.800	1.174	0.338-4.073
Treatment	2.385	1.083	4.846	0.028	10.856	1.299-90.739

At the six-month follow-up, two out of 12 patients (16.7%) in the open reduction group and one out of 14 (7.1%) in the closed reduction group reported occlusal disturbances. No patients reported pain or reduced chewing function, and there were no cases of facial nerve weakness or mandibular nerve sensitivity deficits at the six-month follow-up.

## Discussion

Regarding the fracture side distribution of MCFs, the present study observed a predominance of unilateral fractures (82.1%), which aligns with findings in other pediatric trauma studies, reporting unilateral MCF frequencies ranging from 71.4% to 83% [3,28,29].

In this study, 42.9% had concomitant single mandibular fractures, with the symphysis being the most commonly affected region, followed by the body, and 14.3% had multiple fractures. Symphyseal fractures are related and exist along with condylar fractures; whenever there is an anterior blow to the mandible, the area of impact acts like a lever resulting in condylar fractures [30,31]. These findings correspond with other studies, where the symphyseal region was also the most common additional fracture site [3,28,32]. However, in contrast, Thorén et al. [33] reported only three additional fractures in a sample of 138 children with condylar fractures.

Additionally, the study found that 64% of the pediatric patients have MCFs which is consistent with the male predominance observed in previous studies, with male prevalence ranging from 64.6% to 77.6% [27,28,32]. This high rate of male subjects has been attributed to the increased involvement of males in sports, violence, and traffic accidents, which are more likely to result in MCFs [34].

There remains significant controversy surrounding the choice between closed and open reduction in treating MCFs in children and adolescents [6,7,35]. The effectiveness of treatment modalities has not been consistently correlated with specific clinical and CT imaging-based parameters of MCFs, i.e., treatment decisions continue to rely on factors such as the cost-risk benefit ratio, clinician experience, degree of invasiveness, and clinical judgment. The current study, through its multivariate approach, aimed to address the role of clinical and radiological parameters in defining functional outcomes in pediatric MCF management. The results indicated that closed reduction was associated with improved functional outcomes in terms of vertical and lateral mandibular ROM, suggesting that for Class I-III fractures with a fracture fragment angulation between 0° and 45°, closed reduction may promote better outcomes. This supports the notion that, due to the high osteogenic potential of the periosteum in the developing craniofacial skeleton, closed treatment of moderately displaced condylar fractures may allow for adequate condylar regeneration, remodeling, and functional restitution of the TMJ [36,37].

Moreover, the present study used mandibular ROM in vertical, lateral, and protrusive directions as the primary outcome measure, referencing distribution-based values for children and adolescents to differentiate between "normal" and "limited" mandibular motion [24-27]. This method of evaluating ROM provides more nuanced data, expressed in percentile ranks, accounting for sex and age, which is an improvement over simply categorizing ROM as above or below a cut-off. Future research is warranted to establish a consensus on measurable outcomes in pediatric MCF management to allow for consistent comparisons across trials [6]. 

One of the key findings of the study was the potential utility of CBCT in diagnosing specific subtypes of MCFs. This technique may be especially helpful in distinguishing fractures that might be difficult to detect in two-dimensional imaging techniques, such as panoramic radiography [38]. CBCT offers several advantages over traditional CT scans, including a smaller footprint, lower power requirement, reduced cost, lower radiation doses, and faster scan times [9,13,39]. However, its use should be balanced against the potential for higher radiation exposure compared to digital panoramic radiographs [40,41]. Future studies may need to evaluate whether CBCT can be reliably integrated into clinical decision-making, especially when combined with clinical history, physical examination, and standard radiographic evaluation.

Also worth noting is the relatively short follow-up period in this study. Long-term outcomes, such as growth disturbances, functional impairments, and malocclusion, are important considerations in the treatment of pediatric MCFs. Previous studies have indicated that reduced growth and remodeling capacities in the MCF region can lead to facial asymmetries and malocclusion, especially in displaced fractures [42-44]. Long-term follow-ups may reveal the impact of such fractures on the developing craniofacial skeleton, which could be influenced by the patient's age at the time of injury and the extent of fracture displacement [45,46]. These findings suggest that further studies should be encouraged to explore the remodeling potential in displaced MCFs, particularly in adolescents where the potential for complete remodeling is lower [47,48].

The study does have several limitations, the most notable being its retrospective design. Randomized controlled trials with more detailed protocols are needed to refine treatment strategies for pediatric MCFs. Additionally, the study did not fully explore the effects of different fracture patterns or cases with additional maxillofacial fractures, which may provide further insight into functional outcomes. Furthermore, potential rater bias in the assessment of CBCT images is a concern. Future studies should consider incorporating multiple observers to ensure the reliability and validity of imaging assessments in multicenter settings.

## Conclusions

This preliminary study suggests a correlation between treatment modality and functional outcomes at the six-month follow-up. ORIF of subcondylar fractures in children and adolescents may carry a higher risk of poor functional outcomes. The findings support closed reduction as the preferred approach for Class I-III cases with subcondylar fracture fragments angulated between 0° and 45°.

## References

[1] Alcalá-Galiano A, Arribas-García IJ, Martín-Pérez MA, Romance A, Montalvo-Moreno JJ, Juncos JM (2008). Pediatric facial fractures: children are not just small adults. Radiographics.

[2] Smartt JM Jr, Low DW, Bartlett SP (2005). The pediatric mandible: I. A primer on growth and development. Plast Reconstr Surg.

[3] Choi J, Oh N, Kim IK (2005). A follow-up study of condyle fracture in children. Int J Oral Maxillofac Surg.

[4] Deleyiannis FW, Vecchione L, Martin B, Jiang S, Sotereanos G (2006). Open reduction and internal fixation of dislocated condylar fractures in children: long-term clinical and radiologic outcomes. Ann Plast Surg.

[5] Proffit WR, Vig KW, Turvey TA (1980). Early fracture of the mandibular condyles: frequently an unsuspected cause of growth disturbances. Am J Orthod.

[6] Jenkyn I, Bosley R, Jenkyn C, Basyuni S, Fowell C (2023). Management of mandibular condyle fractures in paediatric patients: a systematic review. J Oral Maxillofac Res.

[7] Esposito NR, Cisternas IN, Gonzalez AC (2024). Surgical treatment of paediatric fractures of the mandibular condyle: a systematic review of the literature. Br J Oral Maxillofac Surg.

[8] Villarreal PM, Monje F, Junquera LM, Mateo J, Morillo AJ, González C (2004). Mandibular condyle fractures: determinants of treatment and outcome. J Oral Maxillofac Surg.

[9] Scarfe WC (2005). Imaging of maxillofacial trauma: evolutions and emerging revolutions. Oral Surg Oral Med Oral Pathol Oral Radiol Endod.

[10] Ziegler CM, Woertche R, Brief J, Hassfeld S (2002). Clinical indications for digital volume tomography in oral and maxillofacial surgery. Dentomaxillofac Radiol.

[11] Sirin Y, Guven K, Horasan S, Sencan S (2010). Diagnostic accuracy of cone beam computed tomography and conventional multislice spiral tomography in sheep mandibular condyle fractures. Dentomaxillofac Radiol.

[12] Rashid A, Feinberg L, Fan K (2024). The application of cone beam computed tomography (CBCT) on the diagnosis and management of maxillofacial trauma. Diagnostics (Basel).

[13] Venkatesh E, Elluru SV (2017). Cone beam computed tomography: basics and applications in dentistry. J Istanb Univ Fac Dent.

[14] Dreizin D, Nam AJ, Tirada N (2016). Multidetector CT of mandibular fractures, reductions, and complications: a clinically relevant primer for the radiologist. Radiographics.

[15] He D, Yang C, Chen M, Jiang B, Wang B (2009). Intracapsular condylar fracture of the mandible: our classification and open treatment experience. J Oral Maxillofac Surg.

[16] Zhou Z, Li Z, Ren J, He M, Huang Y, Tian W, Tang W (2018). Digital diagnosis and treatment of mandibular condylar fractures based on Extensible Neuro imaging Archive Toolkit (XNAT). PLoS One.

[17] Du C, Xu B, Zhu Y, Zhu M (2021). Radiographic evaluation in three dimensions of condylar fractures with closed treatment in children and adolescents. J Craniomaxillofac Surg.

[18] Li MX, Xing X, Li ZB, Li Z (2021). Classification and treatment strategies for condylar fractures in children. Br J Oral Maxillofac Surg.

[19] Shiyan W, Shi J, Zhang W (2024). Effect of different treatment modalities for condylar fractures in childhood on mandibular symmetry and temporomandibular joint function: a retrospective study. J Craniofac Surg.

[20] Bhagol A, Singh V, Kumar I, Verma A (2011). Prospective evaluation of a new classification system for the management of mandibular subcondylar fractures. J Oral Maxillofac Surg.

[21] Vidhya V, Dominic S, Mohan S, Raneesh KE (2023). Outcome of management of mandibular sub condylar fractures based on classification system using cone-beam computed tomography. J Maxillofac Oral Surg.

[22] Chang SP, Yang Y, Shi LQ, Liu YW, Liu Y, Ma Q (2018). Modification of the measurement of the major variables in mandibular condylar fractures: angulation of sidewards displacement and shortening of the height of the ramus. Br J Oral Maxillofac Surg.

[23] Dworkin SF, LeResche L (1992). Research diagnostic criteria for temporomandibular disorders: review, criteria, examinations and specifications, critique. J Craniomandib Disord.

[24] Müller L, van Waes H, Langerweger C, Molinari L, Saurenmann RK (2013). Maximal mouth opening capacity: percentiles for healthy children 4-17 years of age. Pediatr Rheumatol Online J.

[25] Stoustrup P, Kristensen KD, Küseler A, Herlin T, Pedersen TK (2016). Normative values for mandibular mobility in Scandinavian individuals 4-17 years of age. J Oral Rehabil.

[26] Iturriaga V, Bornhardt T, Arias A, Antiao M, Aravena Y, Navarro P, Manterola C (2017). Mandibular range movement in pediatric patients. Int J Odontostomat.

[27] Hirsch C, John MT, Lautenschläger C, List T (2006). Mandibular jaw movement capacity in 10-17-yr-old children and adolescents: normative values and the influence of gender, age, and temporomandibular disorders. Eur J Oral Sci.

[28] Lekven N, Neppelberg E, Tornes K (2011). Long-term follow-up of mandibular condylar fractures in children. J Oral Maxillofac Surg.

[29] Landes CA, Day K, Glasl B, Ludwig B, Sader R, Kovács AF (2008). Prospective evaluation of closed treatment of nondisplaced and nondislocated mandibular condyle fractures versus open reposition and rigid fixation of displaced and dislocated fractures in children. J Oral Maxillofac Surg.

[30] Nayak SS, Arun S, Taranath Kamath A, Jaladhigere Lakshmanagowda B, Dubey E, Koshy J (2021). The influence of the mandibular chin angle on the occurrence of mandibular condylar fracture: a retrospective study. ScientificWorldJournal.

[31] Xin P, Jiang B, Dai J, Hu G, Wang X, Xu B, Shen SG (2014). Finite element analysis of type B condylar head fractures and osteosynthesis using two positional screws. J Craniomaxillofac Surg.

[32] Thorén H, Iizuka T, Hallikainen D, Lindqvist C (1998). Radiologic changes of the temporomandibular joint after condylar fractures in childhood. Oral Surg Oral Med Oral Pathol Oral Radiol Endod.

[33] Thorén H, Hallikainen D, Iizuka T, Lindqvist C (2001). Condylar process fractures in children: a follow-up study of fractures with total dislocation of the condyle from the glenoid fossa. J Oral Maxillofac Surg.

[34] Sawazaki R, Lima Júnior SM, Asprino L, Moreira RW, de Moraes M (2010). Incidence and patterns of mandibular condyle fractures. J Oral Maxillofac Surg.

[35] Stähli C, Eliades T, Papageorgiou SN (2021). Functional appliance treatment for mandibular fractures: a systematic review with meta-analyses. J Oral Rehabil.

[36] Chrcanovic BR (2012). Open versus closed reduction: mandibular condylar fractures in children. Oral Maxillofac Surg.

[37] Steed MB, Schadel CM (2017). Management of pediatric and adolescent condylar fractures. Atlas Oral Maxillofac Surg Clin North Am.

[38] Kaeppler G, Cornelius CP, Ehrenfeld M, Mast G (2013). Diagnostic efficacy of cone-beam computed tomography for mandibular fractures. Oral Surg Oral Med Oral Pathol Oral Radiol.

[39] Demehri S, Baffour FI, Klein JG (2023). Musculoskeletal CT imaging: state-of-the-art advancements and future directions. Radiology.

[40] Angelopoulos C, Thomas SL, Hechler S, Parissis N, Hlavacek M (2008). Comparison between digital panoramic radiography and cone-beam computed tomography for the identification of the mandibular canal as part of presurgical dental implant assessment. J Oral Maxillofac Surg.

[41] Lee H, Badal A (2021). A review of doses for dental imaging in 2010-2020 and development of a web dose calculator. Radiol Res Pract.

[42] Wiltfang J, Halling F, Merten HA, Luhr HG (1991). Mandibular condyle fractures in childhood: effects on growth and function [Article in German]. Dtsch Zahnarztl Z.

[43] McGuirt WF, Salisbury PL 3rd (1987). Mandibular fractures. Their effect on growth and dentition. Arch Otolaryngol Head Neck Surg.

[44] Demianczuk AN, Verchere C, Phillips JH (1999). The effect on facial growth of pediatric mandibular fractures. J Craniofac Surg.

[45] Nørholt SE, Krishnan V, Sindet-Pedersen S, Jensen I (1993). Pediatric condylar fractures: a long-term follow-up study of 55 patients. J Oral Maxillofac Surg.

[46] Hovinga J, Boering G, Stegenga B (1999). Long-term results of nonsurgical management of condylar fractures in children. Int J Oral Maxillofac Surg.

[47] Naik P (2021). Remodelling in children's fractures and limits of acceptability. Indian J Orthop.

[48] Staderini E, Patini R, Tepedino M, Gasparini G, Zimbalatti MA, Marradi F, Gallenzi P (2020). Radiographic assessment of pediatric condylar fractures after conservative treatment with functional appliances-a systematic review. Int J Environ Res Public Health.

